# 2-(4-Chloro­phen­yl)-3-ethyl­sulfinyl-5-fluoro-1-benzofuran

**DOI:** 10.1107/S1600536810008172

**Published:** 2010-03-06

**Authors:** Hong Dae Choi, Pil Ja Seo, Byeng Wha Son, Uk Lee

**Affiliations:** a Department of Chemistry, Dongeui University, San 24 Kaya-dong Busanjin-gu, Busan 614-714, Republic of Korea

## Abstract

In the title compound, C_16_H_12_ClFO_2_S, the 4-chloro­phenyl ring is rotated out of the benzofuran plane, as indicated by the dihedral angle of 19.79 (8)°. The crystal structure exhibits weak inter­molecular C—H⋯O hydrogen bonds and C—H⋯π inter­actions.

## Related literature

For the crystal structures of similar 3-ethyl­sulfinyl-2-(4-fluoro­phen­yl)-5-halo-1-benzofuran derivatives, see: Choi *et al.* (2010**a* ▶,*b* ▶,c* ▶). For the pharmacological activity of benzofuran compounds, see: Aslam *et al.* (2006 ▶); Galal *et al.* (2009 ▶); Khan *et al.* (2005 ▶). For natural products with benzofuran rings, see: Akgul & Anil (2003 ▶); Soekamto *et al.* (2003 ▶).
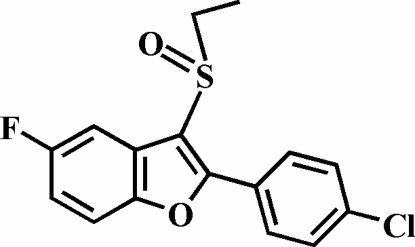


## Experimental

### 

#### Crystal data

C_16_H_12_ClFO_2_S
*M* *_r_* = 322.77
Monoclinic, 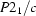

*a* = 11.5257 (9) Å
*b* = 7.9655 (6) Å
*c* = 16.395 (1) Å
β = 106.518 (1)°
*V* = 1443.07 (18) Å^3^
*Z* = 4
Mo *K*α radiation
μ = 0.42 mm^−1^
*T* = 173 K
0.40 × 0.35 × 0.35 mm

#### Data collection

Bruker SMART APEXII CCD diffractometer
Absorption correction: multi-scan (*SADABS*; Bruker, 2009 ▶) *T*_min_ = 0.850, *T*_max_ = 0.867
12345 measured reflections
3290 independent reflections
2713 reflections with *I* > 2σ(*I*)
*R*_int_ = 0.028

#### Refinement

*R*[*F* ^2^ > 2σ(*F* ^2^)] = 0.034
*wR*(*F* ^2^) = 0.085
*S* = 1.05
3290 reflections
191 parameters
H-atom parameters constrained
Δρ_max_ = 0.29 e Å^−3^
Δρ_min_ = −0.27 e Å^−3^

### 

Data collection: *APEX2* (Bruker, 2009 ▶); cell refinement: *SAINT* (Bruker, 2009 ▶); data reduction: *SAINT*; program(s) used to solve structure: *SHELXS97* (Sheldrick, 2008 ▶); program(s) used to refine structure: *SHELXL97* (Sheldrick, 2008 ▶); molecular graphics: *ORTEP-3* (Farrugia, 1997 ▶) and *DIAMOND* (Brandenburg, 1998 ▶); software used to prepare material for publication: *SHELXL97*.

## Supplementary Material

Crystal structure: contains datablocks global, I. DOI: 10.1107/S1600536810008172/bh2275sup1.cif

Structure factors: contains datablocks I. DOI: 10.1107/S1600536810008172/bh2275Isup2.hkl

Additional supplementary materials:  crystallographic information; 3D view; checkCIF report

## Figures and Tables

**Table 1:** Hydrogen-bond geometry (Å, °) *Cg* is the centroid of the C9–C14 4-chloro­phenyl ring.

*D*—H⋯*A*	*D*—H	H⋯*A*	*D*⋯*A*	*D*—H⋯*A*
C6—H6⋯O2^i^	0.93	2.52	3.371 (2)	152
C11—H11⋯O2^ii^	0.93	2.53	3.116 (2)	121
C15—H15*A*⋯*Cg*^ii^	0.97	2.66	3.560 (2)	155

Symmetry codes: (i) 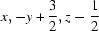; (ii) 
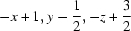
.

**Fig. 1.**
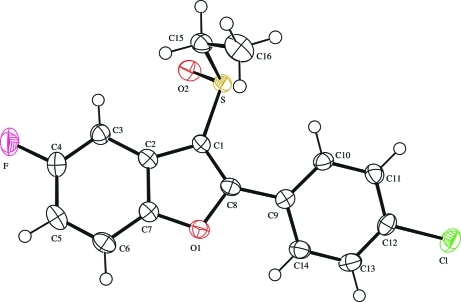
The molecular structure of the title compound with the atom numbering scheme. Displacement ellipsoids are drawn at the 50 % probability level. H atoms are presented as small spheres of arbitrary radius.

**Fig. 2.**
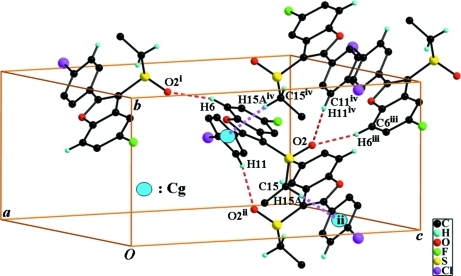
C—H···O and C—H···π interactions (dotted lines) in the crystal structure of the title compound. Cg denotes the ring centroid. [Symmetry codes: (i) x, - y + 3/2, z - 1/2; (ii) - x + 1, y - 1/2, - z + 3/2; (iii) x, - y + 3/2, z + 1/2; (iv) - x + 1, y + 1/2, - z + 3/2.]

## supplementary crystallographic information

### Comment

Compounds containing benzofuran moiety show diverse pharmacological activities such as antifungal (Aslam *et al.*, 2006), antitumor and antiviral (Galal *et al.*, 2009), antimicrobial (Khan *et al.*, 2005) properties. These compounds are widely occurring in nature (Akgul & Anil, 2003; Soekamto *et al.*, 2003). As a part of our ongoing studies of the effect of side chain substituents on the solid state structures of 3-ethylsulfinyl-2-(4-fluorophenyl)-5-halo-1-benzofuran analogues (Choi *et al.*, 2010*a*,*b*,*c*), we report the crystal structure of the title compound (Fig. 1).

The benzofuran unit is essentially planar, with a mean deviation of 0.012 (1) Å from the least-squares plane defined by the nine constituent atoms. The dihedral angle formed by the benzofuran plane and the 4-fluorophenyl ring is 19.79 (8)°. The crystal packing (Fig. 2) is stabilized by two different C—H···O hydrogen bonds; the first between the benzene H atom and the oxygen of the S═O unit, with a C6—H6···O2^i^, and the second between the 4-chlorophenyl H atom and the oxygen of the S═O unit, with a C11—H11···O2^ii^, respectively (Table 1). The crystal packing (Fig. 2) is further stabilized by a C—H···π interaction between the methylene H atom and the 4-chlorophenyl ring, with a C15—H15A···Cg^ii^ (Table 1; Cg is the centroid of the C9–C14 4-chlorophenyl ring).

### Experimental

77% 3-Chloroperoxybenzoic acid (291 mg, 1.3 mmol) was added in small portions to a stirred solution of 2-(4-chlorophenyl)-3-ethylsulfanyl-5-fluoro-1-benzofuran (368 mg, 1.2 mmol) in dichloromethane (30 mL) at 273 K. After being stirred at room temperature for 4h, the mixture was washed with saturated sodium bicarbonate solution and the organic layer was separated, dried over magnesium sulfate, filtered and concentrated at reduced pressure. The residue was purified by column chromatography (hexane–ethyl acetate, 1:1 *v*/*v*) to afford the title compound as a colorless solid [yield 79%, m.p. 405–406 K; *R*_f_ = 0.64 (hexane–ethyl acetate, 1:1 *v*/*v*)]. Single crystals suitable for X-ray diffraction were prepared by slow evaporation of a solution of the title compound in acetone at room temperature.

### Refinement

All H atoms were positioned geometrically and refined using a riding model, with C—H = 0.93 Å for aryl, 0.97 Å for methylene, and 0.96 Å for methyl H atoms. *U*_iso_(H) = 1.2*U*_eq_(C) for aryl and methylene H atoms, and 1.5*U*_eq_(C) for methyl H atoms.

### Crystal data

**Table:**

C_16_H_12_ClFO_2_S	*F*(000) = 664
*M**_r_* = 322.77	*D*_x_ = 1.486 Mg m^−^^3^
Monoclinic, *P*2_1_/*c*	Melting point = 405–406 K
Hall symbol: -P 2ybc	Mo *K*α radiation, λ = 0.71073 Å
*a* = 11.5257 (9) Å	Cell parameters from 5508 reflections
*b* = 7.9655 (6) Å	θ = 2.6–27.4°
*c* = 16.395 (1) Å	µ = 0.42 mm^−^^1^
β = 106.518 (1)°	*T* = 173 K
*V* = 1443.07 (18) Å^3^	Block, colourless
*Z* = 4	0.40 × 0.35 × 0.35 mm

### Data collection

**Table:**

Bruker SMART APEXII CCD diffractometer	3290 independent reflections
Radiation source: Rotating Anode	2713 reflections with *I* > 2σ(*I*)
Bruker HELIOS graded multilayer optics	*R*_int_ = 0.028
Detector resolution: 10.0 pixels mm^-1^	θ_max_ = 27.5°, θ_min_ = 1.8°
φ and ω scans	*h* = −14→14
Absorption correction: multi-scan (*SADABS*; Bruker, 2009)	*k* = −10→10
*T*_min_ = 0.850, *T*_max_ = 0.867	*l* = −21→20
12345 measured reflections	

### Refinement

**Table:**

Refinement on *F*^2^	Primary atom site location: structure-invariant direct methods
Least-squares matrix: full	Secondary atom site location: difference Fourier map
*R*[*F*^2^ > 2σ(*F*^2^)] = 0.034	Hydrogen site location: difference Fourier map
*wR*(*F*^2^) = 0.085	H-atom parameters constrained
*S* = 1.05	*w* = 1/[σ^2^(*F*_o_^2^) + (0.0338*P*)^2^ + 0.7418*P*] where *P* = (*F*_o_^2^ + 2*F*_c_^2^)/3
3290 reflections	(Δ/σ)_max_ < 0.001
191 parameters	Δρ_max_ = 0.29 e Å^−^^3^
0 restraints	Δρ_min_ = −0.27 e Å^−^^3^
0 constraints	

### Fractional atomic coordinates and isotropic or equivalent isotropic displacement parameters (Å^2^)

**Table:**

	*x*	*y*	*z*	*U*_iso_*/*U*_eq_	
Cl	1.01429 (4)	0.47181 (6)	0.72194 (3)	0.03651 (13)	
S	0.37916 (4)	0.44605 (5)	0.71678 (2)	0.02292 (11)	
F	−0.02836 (9)	0.79231 (16)	0.49298 (7)	0.0447 (3)	
O1	0.45081 (10)	0.71492 (14)	0.53223 (7)	0.0262 (3)	
O2	0.30653 (12)	0.54333 (16)	0.76279 (8)	0.0335 (3)	
C1	0.38345 (15)	0.56819 (19)	0.62737 (10)	0.0228 (3)	
C2	0.28142 (15)	0.6562 (2)	0.57188 (10)	0.0242 (3)	
C3	0.15813 (15)	0.6723 (2)	0.56551 (11)	0.0282 (4)	
H3	0.1231	0.6178	0.6028	0.034*	
C4	0.09200 (16)	0.7732 (2)	0.50110 (12)	0.0321 (4)	
C5	0.13881 (17)	0.8572 (2)	0.44327 (11)	0.0328 (4)	
H5	0.0886	0.9230	0.4008	0.039*	
C6	0.26012 (17)	0.8427 (2)	0.44906 (11)	0.0292 (4)	
H6	0.2942	0.8969	0.4112	0.035*	
C7	0.32842 (15)	0.7429 (2)	0.51444 (10)	0.0249 (3)	
C8	0.48285 (15)	0.6093 (2)	0.60191 (10)	0.0233 (3)	
C9	0.61217 (15)	0.5719 (2)	0.63190 (10)	0.0236 (3)	
C10	0.65770 (15)	0.4350 (2)	0.68435 (11)	0.0262 (4)	
H10	0.6045	0.3628	0.7002	0.031*	
C11	0.78105 (15)	0.4052 (2)	0.71314 (11)	0.0274 (4)	
H11	0.8108	0.3149	0.7489	0.033*	
C12	0.85925 (15)	0.5115 (2)	0.68801 (11)	0.0270 (4)	
C13	0.81697 (16)	0.6470 (2)	0.63528 (11)	0.0308 (4)	
H13	0.8706	0.7166	0.6183	0.037*	
C14	0.69410 (16)	0.6776 (2)	0.60811 (11)	0.0288 (4)	
H14	0.6653	0.7698	0.5735	0.035*	
C15	0.28229 (16)	0.2801 (2)	0.66061 (11)	0.0285 (4)	
H15A	0.2502	0.2179	0.7002	0.034*	
H15B	0.2146	0.3287	0.6177	0.034*	
C16	0.3499 (2)	0.1613 (2)	0.61849 (13)	0.0414 (5)	
H16A	0.3808	0.2223	0.5786	0.062*	
H16B	0.2959	0.0750	0.5891	0.062*	
H16C	0.4159	0.1113	0.6610	0.062*	

### Atomic displacement parameters (Å^2^)

**Table:**

	*U*^11^	*U*^22^	*U*^33^	*U*^12^	*U*^13^	*U*^23^
Cl	0.0231 (2)	0.0483 (3)	0.0378 (3)	0.00038 (19)	0.00815 (18)	−0.0005 (2)
S	0.0255 (2)	0.0234 (2)	0.0200 (2)	0.00029 (16)	0.00665 (15)	0.00033 (15)
F	0.0275 (6)	0.0575 (8)	0.0458 (7)	0.0124 (5)	0.0052 (5)	0.0073 (6)
O1	0.0286 (6)	0.0259 (6)	0.0249 (6)	0.0006 (5)	0.0091 (5)	0.0030 (5)
O2	0.0433 (8)	0.0313 (7)	0.0311 (7)	0.0041 (6)	0.0192 (6)	−0.0023 (5)
C1	0.0251 (8)	0.0207 (8)	0.0223 (8)	0.0015 (6)	0.0065 (6)	0.0010 (6)
C2	0.0293 (9)	0.0213 (8)	0.0218 (8)	0.0021 (7)	0.0072 (7)	−0.0013 (6)
C3	0.0279 (9)	0.0302 (9)	0.0269 (9)	0.0025 (7)	0.0085 (7)	0.0007 (7)
C4	0.0275 (9)	0.0348 (10)	0.0315 (9)	0.0059 (8)	0.0043 (7)	−0.0031 (8)
C5	0.0400 (11)	0.0291 (9)	0.0246 (9)	0.0081 (8)	0.0017 (8)	0.0012 (7)
C6	0.0407 (10)	0.0249 (9)	0.0218 (8)	0.0019 (7)	0.0086 (7)	0.0001 (7)
C7	0.0284 (9)	0.0226 (8)	0.0240 (8)	0.0017 (7)	0.0077 (7)	−0.0024 (6)
C8	0.0292 (9)	0.0191 (8)	0.0218 (8)	−0.0012 (7)	0.0077 (7)	−0.0008 (6)
C9	0.0254 (8)	0.0234 (8)	0.0227 (8)	−0.0014 (6)	0.0080 (7)	−0.0040 (6)
C10	0.0264 (9)	0.0224 (8)	0.0322 (9)	−0.0027 (7)	0.0123 (7)	−0.0007 (7)
C11	0.0290 (9)	0.0233 (8)	0.0300 (9)	0.0030 (7)	0.0086 (7)	0.0006 (7)
C12	0.0221 (8)	0.0329 (9)	0.0260 (8)	−0.0008 (7)	0.0070 (7)	−0.0068 (7)
C13	0.0303 (9)	0.0343 (10)	0.0298 (9)	−0.0074 (8)	0.0115 (8)	0.0017 (8)
C14	0.0313 (9)	0.0293 (9)	0.0258 (9)	−0.0029 (7)	0.0082 (7)	0.0037 (7)
C15	0.0308 (9)	0.0234 (8)	0.0286 (9)	−0.0050 (7)	0.0038 (7)	0.0005 (7)
C16	0.0585 (13)	0.0296 (10)	0.0344 (11)	0.0016 (9)	0.0105 (9)	−0.0066 (8)

### Geometric parameters (Å, °)

**Table:**

Cl—C12	1.7427 (17)	C8—C9	1.461 (2)
S—O2	1.4930 (12)	C9—C10	1.395 (2)
S—C1	1.7717 (16)	C9—C14	1.401 (2)
S—C15	1.8047 (17)	C10—C11	1.385 (2)
F—C4	1.364 (2)	C10—H10	0.9300
O1—C7	1.375 (2)	C11—C12	1.382 (2)
O1—C8	1.3820 (19)	C11—H11	0.9300
C1—C8	1.366 (2)	C12—C13	1.382 (2)
C1—C2	1.447 (2)	C13—C14	1.380 (2)
C2—C7	1.395 (2)	C13—H13	0.9300
C2—C3	1.401 (2)	C14—H14	0.9300
C3—C4	1.373 (2)	C15—C16	1.512 (3)
C3—H3	0.9300	C15—H15A	0.9700
C4—C5	1.389 (3)	C15—H15B	0.9700
C5—C6	1.379 (3)	C16—H16A	0.9600
C5—H5	0.9300	C16—H16B	0.9600
C6—C7	1.386 (2)	C16—H16C	0.9600
C6—H6	0.9300		
			
O2—S—C1	106.53 (7)	C10—C9—C8	122.36 (15)
O2—S—C15	106.47 (8)	C14—C9—C8	119.21 (15)
C1—S—C15	97.97 (8)	C11—C10—C9	120.90 (16)
C7—O1—C8	106.91 (12)	C11—C10—H10	119.5
C8—C1—C2	107.11 (14)	C9—C10—H10	119.5
C8—C1—S	127.41 (13)	C12—C11—C10	119.14 (16)
C2—C1—S	125.26 (12)	C12—C11—H11	120.4
C7—C2—C3	118.97 (15)	C10—C11—H11	120.4
C7—C2—C1	105.19 (14)	C11—C12—C13	121.38 (16)
C3—C2—C1	135.83 (16)	C11—C12—Cl	119.38 (14)
C4—C3—C2	116.15 (16)	C13—C12—Cl	119.23 (14)
C4—C3—H3	121.9	C14—C13—C12	119.14 (16)
C2—C3—H3	121.9	C14—C13—H13	120.4
F—C4—C3	117.85 (16)	C12—C13—H13	120.4
F—C4—C5	117.48 (16)	C13—C14—C9	121.00 (16)
C3—C4—C5	124.67 (17)	C13—C14—H14	119.5
C6—C5—C4	119.71 (16)	C9—C14—H14	119.5
C6—C5—H5	120.1	C16—C15—S	111.53 (13)
C4—C5—H5	120.1	C16—C15—H15A	109.3
C5—C6—C7	116.30 (16)	S—C15—H15A	109.3
C5—C6—H6	121.8	C16—C15—H15B	109.3
C7—C6—H6	121.8	S—C15—H15B	109.3
O1—C7—C6	125.43 (15)	H15A—C15—H15B	108.0
O1—C7—C2	110.38 (14)	C15—C16—H16A	109.5
C6—C7—C2	124.18 (16)	C15—C16—H16B	109.5
C1—C8—O1	110.38 (14)	H16A—C16—H16B	109.5
C1—C8—C9	135.43 (15)	C15—C16—H16C	109.5
O1—C8—C9	114.16 (14)	H16A—C16—H16C	109.5
C10—C9—C14	118.43 (16)	H16B—C16—H16C	109.5
			
O2—S—C1—C8	130.71 (15)	C2—C1—C8—O1	−1.44 (18)
C15—S—C1—C8	−119.40 (16)	S—C1—C8—O1	−176.28 (11)
O2—S—C1—C2	−43.25 (16)	C2—C1—C8—C9	176.59 (17)
C15—S—C1—C2	66.64 (15)	S—C1—C8—C9	1.8 (3)
C8—C1—C2—C7	1.33 (18)	C7—O1—C8—C1	0.97 (17)
S—C1—C2—C7	176.32 (12)	C7—O1—C8—C9	−177.52 (13)
C8—C1—C2—C3	−177.60 (19)	C1—C8—C9—C10	20.4 (3)
S—C1—C2—C3	−2.6 (3)	O1—C8—C9—C10	−161.65 (14)
C7—C2—C3—C4	0.7 (2)	C1—C8—C9—C14	−158.99 (19)
C1—C2—C3—C4	179.52 (18)	O1—C8—C9—C14	19.0 (2)
C2—C3—C4—F	−179.86 (15)	C14—C9—C10—C11	0.8 (2)
C2—C3—C4—C5	0.3 (3)	C8—C9—C10—C11	−178.60 (15)
F—C4—C5—C6	179.66 (16)	C9—C10—C11—C12	−1.2 (3)
C3—C4—C5—C6	−0.5 (3)	C10—C11—C12—C13	0.4 (3)
C4—C5—C6—C7	−0.3 (3)	C10—C11—C12—Cl	−178.48 (13)
C8—O1—C7—C6	179.89 (16)	C11—C12—C13—C14	0.8 (3)
C8—O1—C7—C2	−0.08 (17)	Cl—C12—C13—C14	179.64 (14)
C5—C6—C7—O1	−178.60 (15)	C12—C13—C14—C9	−1.2 (3)
C5—C6—C7—C2	1.4 (3)	C10—C9—C14—C13	0.4 (3)
C3—C2—C7—O1	178.38 (14)	C8—C9—C14—C13	179.81 (16)
C1—C2—C7—O1	−0.77 (18)	O2—S—C15—C16	−174.17 (12)
C3—C2—C7—C6	−1.6 (3)	C1—S—C15—C16	75.90 (14)
C1—C2—C7—C6	179.26 (15)		

### Hydrogen-bond geometry (Å, °)

**Table:**

Cg is the centroid of the C9–C14 4-chlorophenyl ring.

**Table:**

*D*—H···*A*	*D*—H	H···*A*	*D*···*A*	*D*—H···*A*
C6—H6···O2^i^	0.93	2.52	3.371 (2)	152
C11—H11···O2^ii^	0.93	2.53	3.116 (2)	121
C15—H15A···Cg^ii^	0.97	2.66	3.560 (2)	155

Symmetry codes: (i) *x*, −*y*+3/2, *z*−1/2; (ii) −*x*+1, *y*−1/2, −*z*+3/2.
